# Systems Genetics of Hepatic Metabolome Reveals Octopamine as a Target for Non-Alcoholic Fatty Liver Disease Treatment

**DOI:** 10.1038/s41598-019-40153-0

**Published:** 2019-03-06

**Authors:** Francois Brial, Aurélie Le Lay, Lyamine Hedjazi, Tsz Tsang, Jane F. Fearnside, Georg W. Otto, Fawaz Alzaid, Steven P. Wilder, Nicolas Venteclef, Jean-Baptiste Cazier, Jeremy K. Nicholson, Chris Day, Alastair D. Burt, Ivo G. Gut, Mark Lathrop, Marc-Emmanuel Dumas, Dominique Gauguier

**Affiliations:** 1 Sorbonne University, University Paris Descartes, University Paris Diderot, INSERM UMR_S 1138, Cordeliers Research Centre, 75006 Paris, France; 2 Computational and Systems Medicine, Department of Surgery and Cancer, Faculty of Medicine, Imperial College London, London, SW7 2AZ United Kingdom; 3 School of Health and Related Research, The University of Sheffield, 30 Regent Court, Sheffield, S10 2TA United Kingdom; 4 Genetics and Genomic Medicine, University College London Institute of Child Health, 30 Guilford Street, London, WC1N 1EH United Kingdom; 5 The Wellcome Trust Centre for Human Genetics, University of Oxford, Oxford, Oxfordshire OX3 7BN United Kingdom; 6 Centre for Computational Biology, Medical School, University of Birmingham, Birmingham, B15 2TT United Kingdom; 7 The Australian National Phenome Centre, Murdoch University, Perth, WA6150 Australia; 8 Faculty of Medical Sciences, Medical School, Framlington Place, Newcastle upon Tyne, NE2 4HH United Kingdom; 9 Faculty of Health and Medical Sciences, The University of Adelaide, Adelaide, SA 5005 Australia; 10 CNAG-CRG, Centre for Genomic Regulation, Barcelona Institute of Science and Technology (BIST), Baldiri Reixac 4, 08028 Barcelona, Spain; 11 McGill University and Genome Quebec Innovation Centre, 740 Doctor Penfield Avenue, Montreal, QC H3A 0G1 Canada; 12 Present Address: Genomics Plc, King Charles House, Oxford, Park End Street, OX1 1JD United Kingdom

## Abstract

Non-alcoholic fatty liver disease (NAFLD) is often associated with obesity and type 2 diabetes. To disentangle etiological relationships between these conditions and identify genetically-determined metabolites involved in NAFLD processes, we mapped ^1^H nuclear magnetic resonance (NMR) metabolomic and disease-related phenotypes in a mouse F2 cross derived from strains showing resistance (BALB/c) and increased susceptibility (129S6) to these diseases. Quantitative trait locus (QTL) analysis based on single nucleotide polymorphism (SNP) genotypes identified diet responsive QTLs in F2 mice fed control or high fat diet (HFD). In HFD fed F2 mice we mapped on chromosome 18 a QTL regulating liver micro- and macrovesicular steatosis and inflammation, independently from glucose intolerance and adiposity, which was linked to chromosome 4. Linkage analysis of liver metabolomic profiling data identified a QTL for octopamine, which co-localised with the QTL for liver histopathology in the cross. Functional relationship between these two QTLs was validated *in vivo* in mice chronically treated with octopamine, which exhibited reduction in liver histopathology and metabolic benefits, underlining its role as a mechanistic biomarker of fatty liver with potential therapeutic applications.

## Introduction

NAFLD is the most common liver disease, characterized by triglyceride accumulation in the liver^1^. Frequent association of NAFLDand obesity complicates investigations into NAFLD etiology^2^. NAFLD assessment in genetic studies is based on proton magnetic resonance spectroscopy, serum alanine aminotransferase (ALT) and aspartate aminotransferase (AST), and liver histopathology^3^. There are therefore unaddressed fundamental questions regarding etiological relationships between NAFLD and obesity and clinical needs to identify biomarkers and therapeutics for NAFLD. There is no treatment for NAFLD apart from those targeting obesity, dyslipidemia and insulin resistance. In previous studies, we identified mouse strains with susceptibility (129S6) or resistance (BALB/c) to obesity^4^ and NAFLD^5^ induced by high-fat diet (HFD), suggesting strain-specific diet-inducible alleles that can be genetically mapped in experimental crosses.

Here we applied quantitative trait locus (QTL) mapping to dissect out the genetic control of physiological, liver histopathological and metabolomic data in 129S6xBALB/c F2 mice. Through this approach, we disentangled NAFLD from obesity and identified octopamine as a genetically-determined metabolite, colocalising with a NAFLD-specific QTL. We then evaluated the therapeutic potential of octopamine and demonstrate it improves hepatic steatosis and glucose tolerance independently of obesity, opening avenues for octopamine in the treatment of NAFLD.

## Results and Discussion

Adult F2 mice fed HFD for 4 months exhibited glucose intolerance and a general increase in body weight (BW), adiposity index (AI) and fasting glycemia when compared to CHD-fed F2 mice, (Supplementary Table S1). Patterns of correlations between body weight, tissue weight, and glucose homeostasis phenotypes were generally conserved in CHD and HFD-fed F2 mice (Supplementary Fig. S1). In both cohorts, there were positive correlations between glucose regulation phenotypes and body weight and adiposity indices, and only weak correlations between fasting glycemia and glucose tolerance. Variability of the main phenotype categories (BW, AI, fasting glycemia, cumulative glycemia) in F2 mice was increased by fat feeding (Supplementary Fig. S2), which concurs with our previous report of increased phenotype heterogeneity in inbred mice fed HFD^6^. Despite being fed HFD for 15 weeks, a large proportion of mice exhibited phenotype values similar to or even below mean values in age-matched CHD-fed F2 mice. These results underline the strong phenotypic impact of gene x environment interactions and indicate that both susceptibility and relative resistance alleles to HFD-induced obesity segregate in the cross.

Over 70% and 53% of CHD-fed F2 mice showed severe liver microvesicular lesions (grades 3a, 3b, see methods) and evidence of macrovesicular lesions, respectively (Supplementary Fig. S3A). Similarly, 73% and 30% of HFD-fed F2 mice showed evidence of microvesicular lesions (grades 1, 2) and macrovesicular lesions, respectively (Supplementary Fig. S3B). Few HFD-fed F2 mice showed lipid-related inflammatory response, indicating a mild steatohepatitis phenotype. Extensive inflammation (*i.e*. panacinar) was not observed. Evidence of ballooning was observed in only four HFD-fed F2 mice. Given that liver histopathology was based on H&E stained sections, assessment of fibrosis was not made, although we did not observe significant liver scarring. Results from liver histology suggest that liver structural anomalies are relatively mild following 15 weeks of HFD feeding, but we cannot rule out possible phenotype progression with prolonged dietary challenge.

To map the genetic regulation of NAFLD-related phenotypes, liver histopathology grades were used to test for linkage to single nucleotide polymorphism (SNP) markers in CHD- and HFD-fed F2 mice. In CHD-fed mice, no evidence of linkage was found with microvesicular lesions (Supplementary Fig. S4A). Significant linkages to macrovesicular lesions were detected in CHD-fed mice on chromosomes 11 (SNP rs3690511, LOD = 3.4) and 18 (SNP rs13483492, LOD = 5.3; 54 cM) (Supplementary Fig. S4B). 129S6 genotypes at the chromosome 18 QTL were associated with increased macrovesicular scores (Supplementary Fig. S4C).

**Figure 1:**
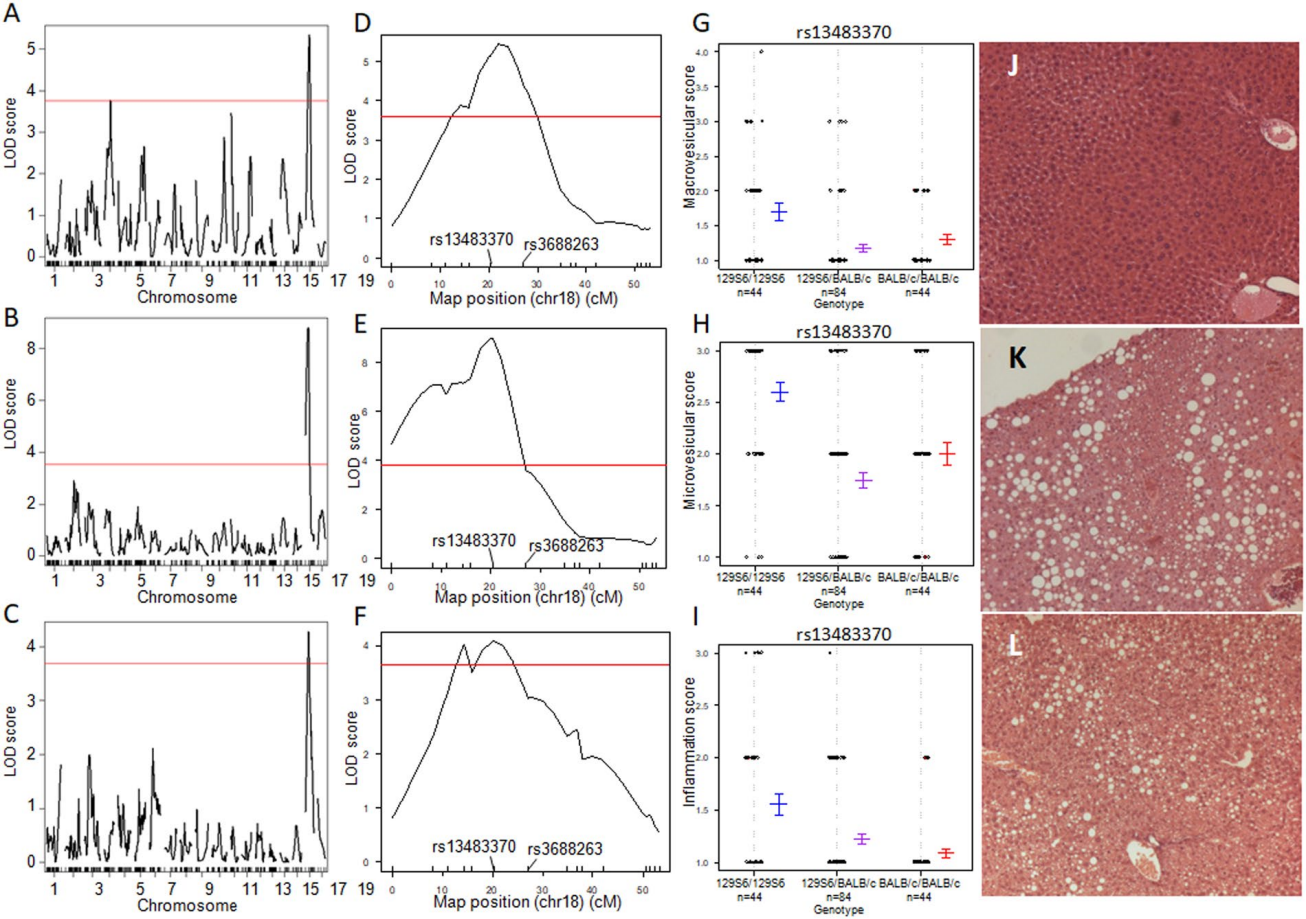
Genome-wide linkage mapping of liver histopathology identifies QTLs for NAFLD on mouse chromosome 18. Linkage mapping data and allele effects are shown for the grades of macrovesicular lesions (**A**,**D**) microvesicular lesions (**B**,**E**) and inflammation (**C**,**F**) in high fat diet (HFD) fed F2 mice. LOD scores are plotted against map distances (centimorgans) across the genome (**A**–**C**) and on chromosome 18 (**D**–**F**). Horizontal red lines indicates statistically significant LOD thresholds (P = 0.001). Effects of genotypes at marker locus rs13483370 on chromosome 18 exhibiting the strongest evidence of linkage to macrovesicular steatosis (**G**), microvesicular steatosis (**H**) and inflammation (**I**) are illustrated by mean values (±SD) of the phenotypes calculated according to the genotype homozygous for the 129S6 or BALB/c alleles, or heterozygous at the locus. Typical examples of liver histopathology in HFD-fed F2 mice homozygous at marker locus rs13483370 for the BALB/c (**J**) or the 129S6 (**K**,**L**) allele are shown (Magnification x50).

In HFD-fed mice, QTLs for the grading of macrovesicular liver lesions were identified on chromosomes 4 (marker locus rs13477976; LOD = 3.8; 38.6 cM) and 18 (marker locus rs13483370; LOD = 5.3; 20.3 cM) (Fig. 1A). Microvesicular lesions (LOD = 9.3) and inflammation (LOD = 4.2) were also consistently linked to the same region of the chromosome 18 QTL (Fig. 1B–F), but proximal to that detected in CHD-fed mice. 129S6 alleles at the locus contributed to increased phenotype severity (Fig. 1G–I).

F2 mice carrying BALB/c homozygous genotypes at the locus rs13483370 showed normal liver histology (Fig. 1J), whereas mice carrying 129S6 homozygous genotypes at the locus showed micro- and macrovesicular steatosis (Fig. 1K) or microvesicular steatosis associated with inflammation (Fig. 1L). In these examples, mice carrying the homozygous 129S6 allele at the marker locus rs13483370, which exhibited evidence of liver histopathology, showed elevated adiposity index (59 × 10^−3^ and 61 × 10^−3^) and liver triglycerides content (118.0 mg/g and 144.4 mg/g) when compared to mice homozygous BALB/c at the locus (adiposity index: 19 × 10^−3^; liver triglycerides: 10.9 mg/g). Linkage to disease status as a binary factor (*i.e*. presence or absence of liver lesions) gave similar results (data not shown). No evidence of significant QTLs was found for ballooning (maximum LOD = 2.04 at marker locus rs13483370 on chromosome 18) or liver necrosis (maximum LOD = 1.66 at marker locus D18Mit202 on chromosome 18) (Supplementary Fig. S5). The rare occurrence of liver ballooning and necrosis in F2 mice may explain the lack of significant QTL for these phenotypes, suggesting that a much larger cross or prolonged HFD feeding may be needed to map their genetic control.

**Figure 2:**
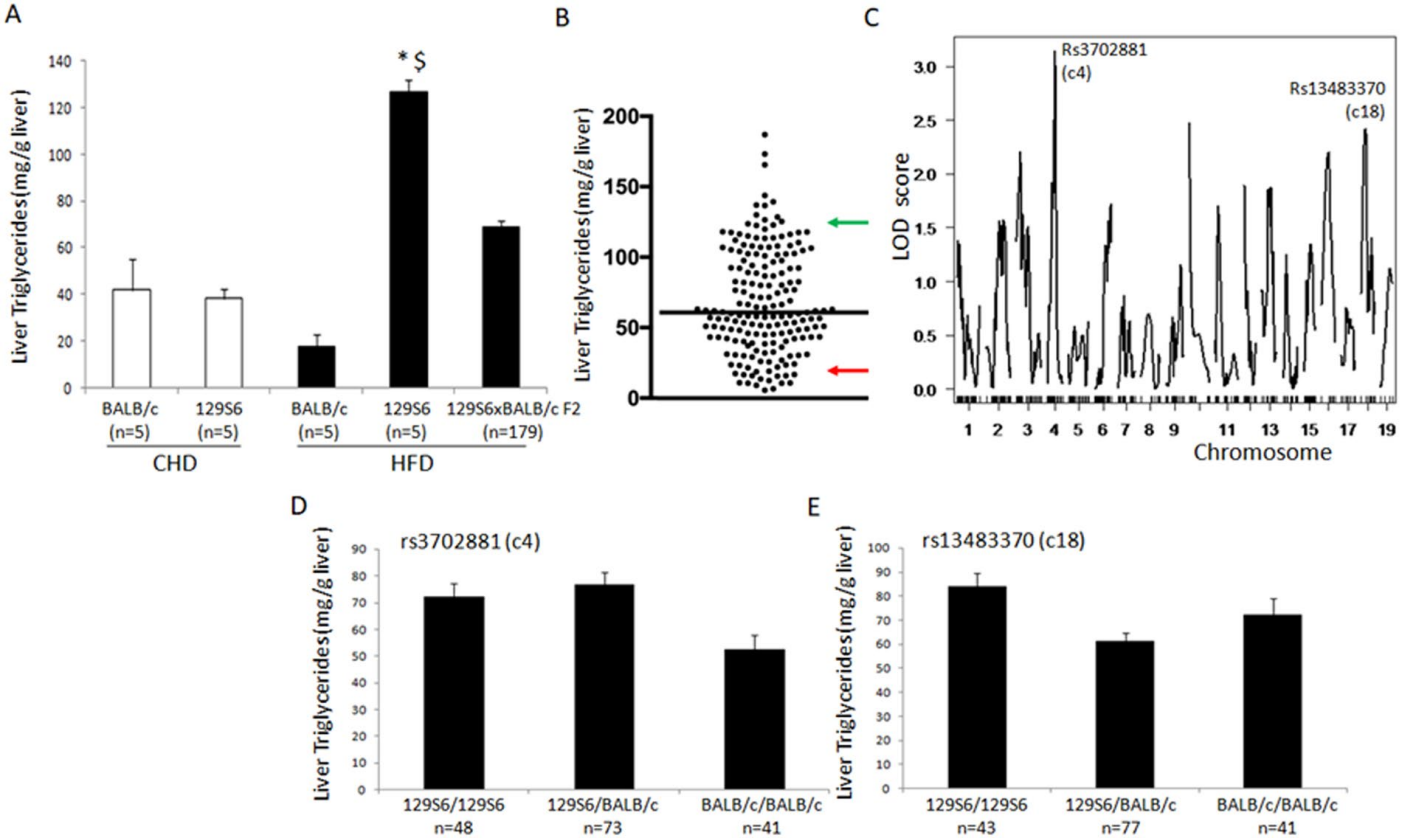
Genetic analysis of liver triglycerides content in 129S6xBALB/c F2 mice fed high fat diet. Concentration of liver triglycerides was determined in BALB/c and 129S6 mice fed control carbohydrate diet (CHD) or high fat diet (HFD) and in 129S6xBALB/c F2 mice fed HFD (**A**). Distribution of liver triglycerides in F2 mice shows the strong variability of the phenotype in the cross (**B**). Solid black line indicates phenotype means in the F2 cohort, and green and red arrows indicate mean values of corresponding phenotypes in 129S6 and BALB/c mice, respectively. Genome-wide scans for liver triglycerides in HFD fed 129S6xBLAB/c F2 mice identified quantitative trait loci of marginal significance on chromosomes 4 and 18 (**C**). LOD scores are plotted against map distances (centimorgans). Effects of genotypes at marker loci rs3702881 (chromosome 4) and rs13483370 (chromosome 18) exhibiting the strongest evidence of linkage to liver triglycerides are illustrated by mean values (±SEM) of the phenotypes calculated according to the genotype homozygous for the 129S6 or BALB/c alleles, or heterozygous at the loci. *P = 0.001 statistically different between 129S6 fed HFD or CHD; ^$^ P = 0.006 statistically different between fat fed 129S6 and fat fed BALB/c.

To further investigate these QTLs beyond histology grades, we determined liver triglycerides content in HFD fed F2 mice and carried out linkage analyses using resulting quantitative values of this phenotype. We verified that 129S6 mice fed HFD showed significantly elevated concentration of liver triglycerides when compared to 129S6 mice fed CHD and BALB/c mice fed HFD, whereas F2 mice showed intermediate values (Fig. 2A) and broad distribution of this phenotype (Fig. 2B). Evidence of marginal linkage to liver triglycerides was detected on chromosomes 4 (rs3702881; LOD = 3.15; 33.4 cM) and 18 (rs13483370; LOD = 2.56; 20.4 cM) (Fig. 2C), thus coinciding with the QTLs linked to microvesicular lesions in the cross. At both QTL, 129S6 were associated with elevated concentration of triglycerides (Fig. 2D,E).

**Figure 3:**
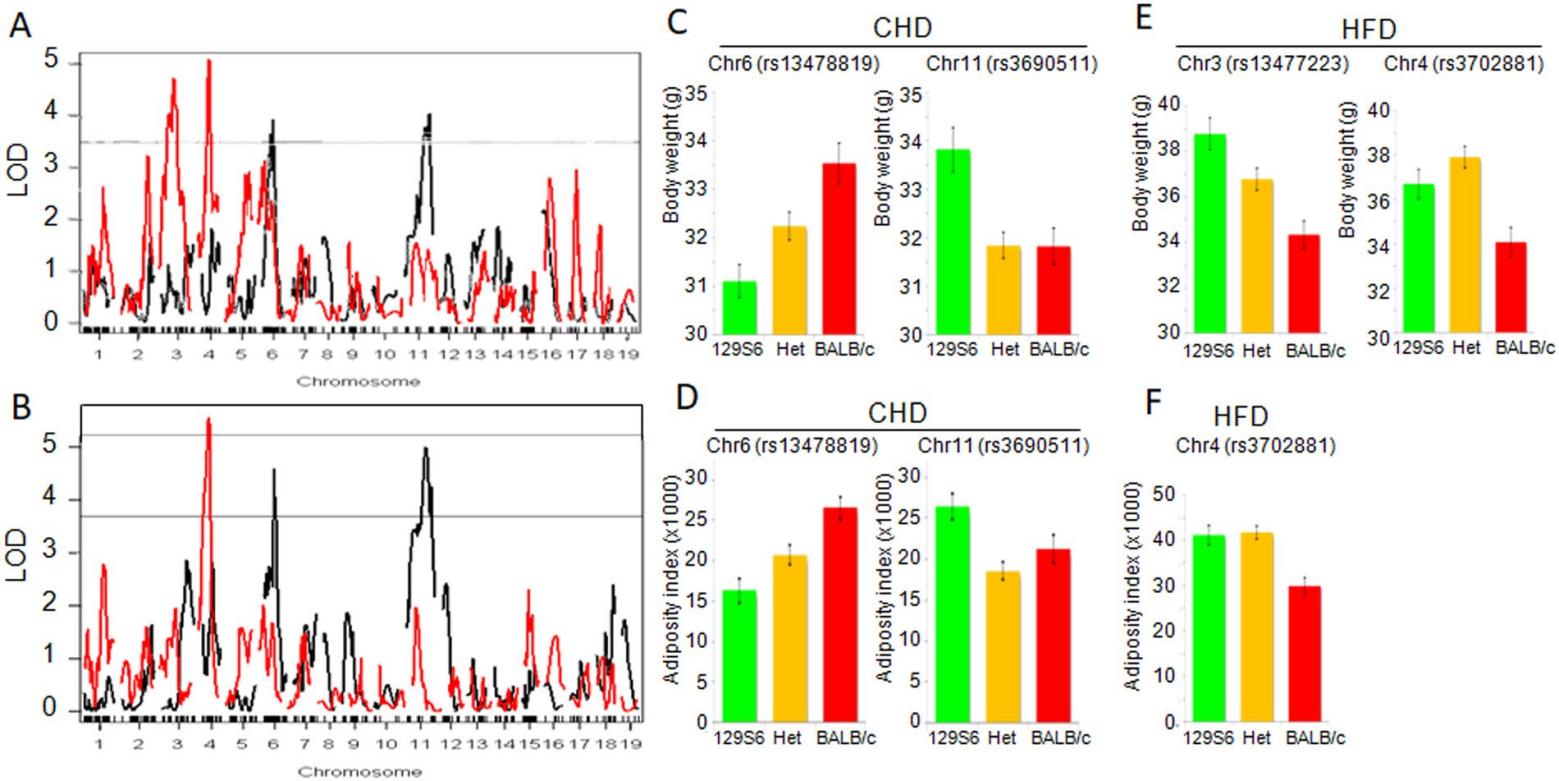
Genome-wide linkage mapping of body weight and adiposity index identifies diet specific genetic control independent of NAFLD. Linkages to body weight (**A**) and adiposity index (**B**) were calculated in (129S6xBALB/c) F2mice fed control diet (CHD) (black lines) or high fat diet (HFD) (red lines). LOD scores are plotted against map distances (centimorgans). Horizontal lines (**A**,**B**) indicate statistically significant LOD thresholds (P = 0.001). The two QTLs on chromosomes 6 and 11 in CHD-fed mice explain a high proportion of the phenotypic variances of BW (10.4–10.7%) and AI (8.8–12.3%). In HFD-fed F2 mice, the QTLs linked to BW explained 13.1–13.7% of the phenotypic variance of BW in the cross. Phenotypic effects of genotypes at markers exhibiting the strongest evidence of linkage are illustrated by phenotype means (±SEM) calculated according to the genotype homozygous for the 129S6 green) or BALB/c allele (red), or heterozygous (orange) at the loci (**C**–**F**). In CHD-fed mice, BALB/c alleles were associated with increased BW and AI on chromosome 6, and decreased BW and AI on chromosome 11 (**C**,**D**), indicating balancing effects of 129S6 and BALB/c alleles at these loci to maintain these phenotypes within similar ranges as observed in the parental strains. In contrast in HFD-fed mice, 129S6 alleles at the QTLs controlling BW (chromosomes 3 and 4) and AI (chromosome 4) were systematically associated with increased BW and AI (**E**,**F**).

To test possible genetic contributions of glucose intolerance and obesity to NAFLD, we carried out linkage analyses to glycemia during the glucose tolerance test, BW and AI in F2 mice fed CHD or HFD. We found little evidence of linkage to glucose regulation in the mouse crosses (maximum LOD = 4.1 for rs13475192 and glycemia 75 minutes after glucose injection in CHD-fed F2 mice) (Supplementary Fig. S6). Two QTLs on chromosomes 6 and 11 were linked to BW (LOD = 4.0 at markers rs13478819 and rs3690511) and AI (LOD = 4.6 at marker rs13478819 and LOD = 5.0 at marker rs3690511) in CHD-fed F2 mice (Fig. 3A–D). These QTLs explain a high proportion of the phenotypic variances of BW (10.4–10.7%) and AI (8.8–12.3%). BALB/c alleles were associated with increased BW and AI on chromosome 6, and decreased BW and AI on chromosome 11 (Fig. 3C,D), indicating balancing effects of 129S6 and BALB/c alleles at these loci to maintain these phenotypes within similar ranges as observed in the parental strains (Supplementary Fig. S2C,D).

In HFD-fed F2 mice, evidence of significant linkage was detected on chromosomes 3 (LOD = 4.7) and 4 (LOD = 5.1) (Fig. 3A) for BW, explaining 13.1–13.7% of the phenotypic variance of BW in the cross. As observed in CHD-fed F2 mice, BW and AI were coordinately controlled by the same locus (rs3702881 on chromosome 4, 33.4 cM, 122.49 Mb) (LOD = 5.6) (Fig. 3B). 129S6 alleles were associated with increased BW and AI (Fig. 3E,F). Lack of linkage to AI on chromosome 3 (Fig. 3B) indicates that different mechanisms regulate BW, presumably through modulation of the weight of other organs than EPD. These data demonstrate the existence of diet-reactive obesity QTLs in the context of a 129S6-BALB/c strain combination and uncover previously unreported obesity QTLs in fat fed mice^7–9^.

These QTL mapping data provided evidence of co-segregation and genetic co-regulation of liver macrovesicular lesions and BW and AI on chromosome 11 (rs3690511) in CHD-fed F2 mice and on chromosome 4 (rs13477976) in HFD-fed F2 mice. These data suggest an effect of a single gene on liver histology through increased adiposity and that liver histopathology may be secondary to obesity caused by diet-reactive genes.

In contrast, the QTL on chromosome 18 linked to macrovesicular lesions in HFD-fed mice was not significantly linked to obesity and glucose tolerance. These results, which show that genetically regulated NAFLD phenotypes do not segregate with obesity and diabetes in the cross are consistent with reports in humans and mice of genetic association to liver fat content regardless of obesity^10,11^.

**Figure 4:**
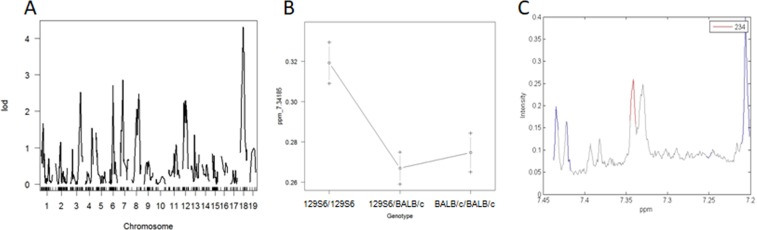
Genome-wide linkage mapping of metabolomic features identifies a metabolite co-regulated with NAFLD. Linkage mapping data and allele effects are shown for the liver metabolomic feature at 7.34185 ppm in high fat diet (HFD) fed F2 mice. LOD scores are plotted against map distances (centimorgans) (**A**). A straight line indicates statistically significant LOD thresholds (P = 0.001). Effects of genotypes at the marker locus on chromosome 18 exhibiting the strongest evidence of linkage to the metabolomic feature 7.34185 ppm are illustrated by mean values (±SD) of the phenotypes calculated according to the genotype homozygous for the 129S6 or BALB/c alleles, or heterozygous at the locus (**B**). ^1^H NMR spectrum from a representative sample revealing the peak pattern between 7.30 and 7.35 ppm is suggestive of octopamine (**C**).

To identify molecular phenotypes specifically contributing to NAFLD, we carried out ^1^H NMR metabolic profiling of liver extracts in HFD-fed F2 mice. Spectral peaks were aligned and quantified using methods previously used in the cross^12^ in order to map metabolomic QTLs in the mouse genome. We mapped 49 QTLs significantly linked to untargeted metabolic features (Supplementary Table S2), including a QTL on chromosome 18 (25 cM) linked to a metabolite at 7.34 ppm, which maps in the close vicinity to the QTLs for liver histopathology and inflammation (Fig. 4A). 129S6 alleles at the locus contributed to increased concentration of the metabolite (Fig. 4B). This feature is part of a doublet at 7.34 ppm (Fig. 4C). Further statistically-aided structural analyses (statistical total correlation spectroscopy, STOCSY) using the 7.34 ppm signal as the driver peak identified other correlated peaks around 6.92 and 3.25 ppm, a pattern characteristic of octopamine.

Octopamine is a norepinephrine analog biosynthesised from tyrosine and a sympatomimetic drug. It can be found in many plant products and seafood. Octopamine stimulates lipolysis^13^ in adipocytes and inhibits glucose uptake^14^. It also stimulates hepatic oxidation of fatty acids^15^. Octopamine and serotonin reduce body fat in *C. elegans*^16^. A molecule related to octopamine (N-*trans*-feruloyloctopamine) shows anti-steatohepatitis properties in a model of NASH^17^.

**Figure 5:**
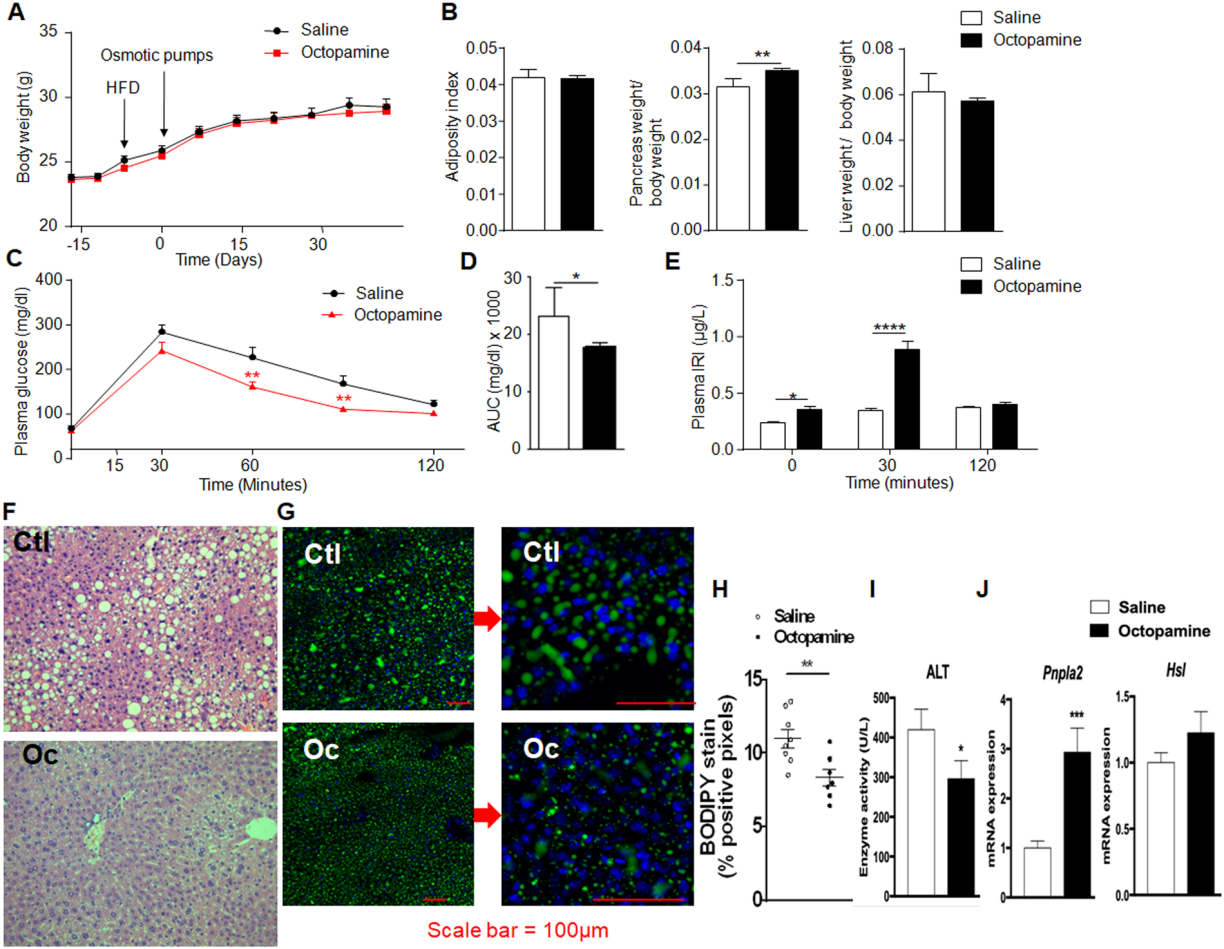
Chronic administration of octopamine in obese mice improves glucose tolerance and reduces liver triglycerides. The effects of 6-week long administration of octopamine (0.125 mg/kg/day) or saline *in vivo* in mice fed high fat diet were tested on body weight (**A**), organ weight (**B**), glucose homeostasis (**C**,**D**), glucose-stimulated insulin secretion (**E**) and liver histopathology (**F**–**H**). AUC was calculated as the sum of plasma glucose values during the IPGTT. Liver sections were stained with haematoxylin and eosin (**F**) or BODIPY (**G**) to quantify fatty acid storage (**H**). Serum alanine aminotransferase (ALT) was determined (**I**) and expression of *Pnpla2* and *Hsl* were analysed by quantitative PCR in liver of octopamine treated mice and saline treated controls (**J**). All measures are from 10 mice per group. Data were analyzed using the unpaired Mann-Whitney test. Results are means ± SEM. *P < 0.05; **P < 0.01; ****P < 0.0001, significantly different to saline treated mice. IRI, immunoreactive insulin; Ctl, control; Oc, octopamine.

Co-localisation of QTLs for NAFLD-related phenotypes and octopamine suggests its possible role in liver histopathology. To test this hypothesis, we used osmotic minipumps inserted subcutaneously to chronically infuse octopamine (0.125 mg/kg/day) in HFD-fed mice over a 42-day period. Octopamine treatment had no effect on body weight, adiposity index and liver weight (Fig. 5A,B), whereas it increased pancreas weight (Fig. 5B), improved glucose tolerance (Fig. 5C,D) and enhanced insulin secretion (Fig. 5E). Histopathology features resembling NAFLD were reduced by octopamine. The most striking observation was the absence of liver macrosteatosis in octopamine-treated mice. Octopamine significantly reduced lipid droplets containing neutral lipids, including triglycerides and cholesterol esters in hepatocytes (Fig. 5G,H), as well as the level of serum alanine aminotransferase (Fig. 5I). Upregulated expression of the gene encoding the patatin-like phospholipase domain containing 2 (*Pnpla2*) in the liver of octopamine treated mice (Fig. 5J) suggests an effect of this metabolite on stimulated lipolysis^18^, which is consistent with the reduction of serum ALT in these mice. In contrast, expression of the hormone-sensitive lipase (*Hsl*) was not affected by the octopamine treatment (Fig. 5J).

**Figure 6:**
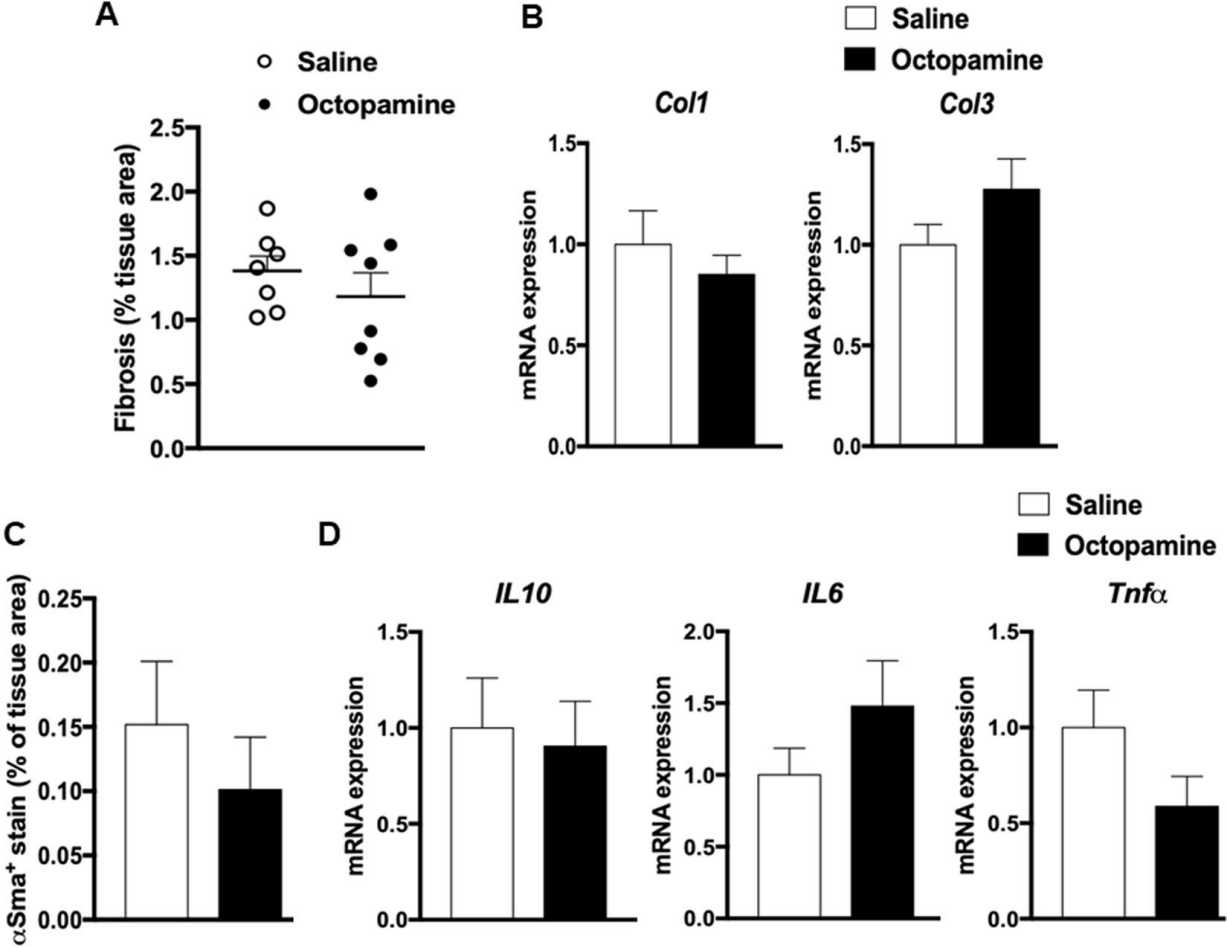
Analysis of liver fibrosis and inflammation in high fat diet fed 129S6 mice chronically treated with octopamine. Data from liver sections stained with red picrosirius or incubated with primary antibody αSMA were used to quantify fibrosis (**A**) and inflammation (**C**), respectively. Each biological replicate represents one slide per animal mounted with at least 3 tissue sections, representing 3 technical replicates, the mean and variance of which is presented as the result per biological replicate. Expression of genes involved in fibrosis (*Col1*, *Col3*) (**B**) or inflammation (*Il6*, *Il10*, *TNF*α) (**D**) were analysed by quantitative PCR in liver of octopamine treated mice and saline treated controls.

To further characterize the effects of octopamine on liver structural and functional phenotypes, we analysed liver collagen accumulation and inflammation in octopamine treated mice and saline infused controls (Fig. 6). Differences in liver collagen and inflammation between octopamine treated mice and controls were not statistically significant (Fig. 6A,C). Liver expression of genes involved in fibrosis (*Col1*, *Col3*) (Fig. 6B) or inflammation (*Il6*, *Il10*, *TNF*α) (Fig. 6D) was not affected by octopamine, even though the coordinated pattern of expression of *Il6* and *TNF*α suggests an impact of octopamine on inflammation. Overall, these results indicate that octopamine has no significant effect on liver fibrosis and inflammation in mice fed HFD for a relatively short period of time, which does not result in severe steatohepatitis but is nevertheless sufficient to induce massive accumulation of triglycerides in the liver. In this context, the effects of octopamine are limited to hepatic lipid metabolism.

By combining metabolomics with systems genetics of disease related variables followed by *in vivo* physiology, we identified co-localized QTLs for hepatic octopamine and NAFLD-related phenotypes, and generated experimental data supporting a role of octopamine in improved glucose tolerance, insulin secretion and liver histopathological features relevant to NAFLD. 129S6 alleles at the chromosome 18 QTL may stimulate octopamine levels as an adaptive mechanism to counteract liver lipid accumulation, as evidenced by the stimulation of *Pnpla2* expression in octopamine treated 129S6 mice. Genetic effects may involve counterintuitive stimulation of hepatic lipases in response to HFD which is consistent with our observation of increased expression of hepatic lipase and monoglyceride lipase in HFD-fed 129S6 mice^19^ and evidence of increased hepatic fat oxidation after prolonged HFD feeding in mice^20^. Liver anomalies are relatively modest in our experimental conditions and prolonged HFD feeding may be required to induce severe liver steatosis. This is supported by our previous report of liver transcriptome regulations in fat fed mice, which showed that expression of genes encoding collagens and interleukins were not affected by HFD feeding in 129S6 mice using the same diet and experimental procedures than in the present study^19^. Knowledge of predominant expression of the octopamine receptor TAAR1 in pancreatic β-cells and its reported effects on stimulation of insulin secretion^21^ provides a functional link to well characterised pathways involving TAAR1 that may explain the beneficial effects of octopamine treatment in obese mice.

In conclusion, our findings contribute to improved fundamental knowledge of NAFLD etiopathogenesis in the context of gene x diet interaction. Even though the size of the cross and the duration of fat feeding may have prevented the detection of more severe histological anomalies, including fibrosis, and the genetic mapping of ballooning, our genetic data shed light on genetic regions carrying genes contributing to early stages of NAFLD. Genetic and physiological data indicating a role of octopamine in reducing liver histopathology, potentially though stimulation of *Pnpla2* expression, may provide therapeutic applications in NAFLD. Further analyses in patients require the development of methods to assay octopamine in human biological specimens. Octopamine being an FDA-approved sympatomimetic drug, it can be easily repurposed for NAFLD treatment, opening therapeutic avenues.

## Methods

### Animals

BALB/c and 129S6 mice were used to produce F1 mice which were sib-mated to derive a cohort of 351 male F2 mice fed carbohydrate (CHD) chow. At 5 weeks, one group of F2 mice (n = 181) was transferred to a 40% high fat diet (HFD) (code 824155; Special Diets Services, Witham, UK). A separate group (n = 148) remained on CHD (code 824153; Special Diets Services, Witham, UK). Composition of the diets is given in Waller Evans *et al*.^22^. Alzet® minipumps (Charles River Lab France, l′Arbresle, France) filled either with octopamine (5.55 mM) (Sigma Aldrich, St Quentin, France) or saline were inserted subcutaneously in 129S6 male mice (Janvier Labs, Courtaboeuf, France) fed HFD (D12492i, Research diets, NJ) for one week.

All animal procedures were carried out in accordance with national and institutional guidelines and regulations. Experiments in F2 mice were carried out in accordance with UK Home Office guidelines on animal welfare under UK Home Office personal and project (PPL1995) licences, and authorized by the animal ethics committee of the University of Oxford. Procedures in 129S6 mice treated with octopamine were carried out under French licence condition (ref. 00486.02) and authorized following review by the ethics committee of the University Pierre and Marie Curie.

### Glucose tolerance

Intraperitoneal glucose tolerance tests (IPGTT) were performed in overnight fasted animals as previously described^4,5^. IPGTT were performed in five months old mice fat fed for 15 weeks (F2) or treated with octopamine for 3 weeks (4 weeks of HFD feeding). Blood glucose was determined (Accucheck, Roche Diagnostics, Welwyn Garden City, UK).

### Tissue sampling

Mice were killed following an overnight fast. Liver and epidydimal fat pads were rapidly removed and blood samples were collected in microvettes CB 300 lithium heparin (Sarstedt, Marnay, France). Plasma was separated and stored at −20 °C until assay. Adiposity index was calculated as the ratio between epidydimal fat pad weight and body weight. Liver samples were collected for histopathology or stored at −80C until metabolomic profiling or gene expression analysis.

### Triglycerides and alanine aminotransferase assays

Liver extracts were prepared and incubated in a buffer containing NP40 (5%) and supernatants containing the triglycerides were separated. Triglycerides concentration was determined on the supernatant fraction using a commercial colorimetric assay according to the manufacturer’s recommendations (Abcam, Paris, France). Triglycerides concentrations were determined by measuring OD at 570 nm. Plasma concentration of alanine aminotransferase (ALT) was measured using a Randox kit (AL 1200, Randox, Roissy en France, France).

### Liver histopathology in 129S6xBALB/c F2 mice

Liver samples from F2 mice and parental 129S6 and BALB/c mice were fixed in formalin and embedded in paraffin. Sections (4 µm) were staining with haematoxylin and eosin (H&E).

In CHD-fed mice, the presence of discrete fat droplets of a size greater than that of the nucleus of a small hepatocyte was used to assess macrovesicular fat with a scoring system (0: none; 1: less than 5 foci and less than 10 cells containing macrovesicular fat within a focus; 2a: less than 5 foci and over 10 cells; 2b: over 5 foci and less than 10 cells; 3: over 5 foci and over 10 cells). Grading scores for microvesicular fat were based on the proportion of affected cells (1: up to 33%; 2: 33–66%; 3a: over 66%; 3b: over 66%, half with foamy appearance at magnification 100x).

In HFD-fed mice, the percentage of affected hepatocytes was used toassess the degree of macrovesicular steatosis on a scale 0–4(0: 0–5%; 1: 6–25%; 2: 26–50%; 3: 51–75%; 4: 76–100%). Microvesicular steatosis was scored on the basis of absence of lesions (0), or focal/indistinct (1) and diffuse (2) lesions identified at low magnification. Ballooning was also assessed (0: none; 1: focal involving small numbers of hepatocytes; 2: extensive involving large numbers of hepatocytes). Ballooning was assessed on the scale of 0–2, 0 being none, 1 being focal involving small numbers of hepatocytes and 2 being extensive involving large numbers of hepatocytes. Inflammation was recorded on a scale 0–2 (0: none; 1: mild focal inflammation; 2: mild/moderate and/or diffuse).

### Liver histology and immunohistochemistry in octopamine treated mice

Tissues were drop-fixed in 4% paraformaldehyde (Sigma-Aldrich, Saint Quentin Fallavier, France) immediately after collection and put through an automated carousel processor for dehydration, clearing and paraffin embedding (Leica, Nanterre, France). Liver sections (6 μm) were mounted on slides (DPX polymerizing mounting medium Sigma-Aldrich, Saint Quentin Fallavier, France) and stained with H&E and BODIPY. Fibrogenesis was assessed by red picrosirius staining (Red 80, Sigma-Aldrich, Saint Quentin Fallavier, France).

For immunohistochemistry analysis, liver sections were quenched with 3% H_2_O_2_, washed with TBS + 0.1% (v/v) Tween-20, blocked with TBS + 3% (w/v) BSA and incubated with diluted primary antibody αSMA (ab124964, Abcam, Paris, France) and then with HRP-conjugated secondary antibody (Bio-Rad, Marnes-la-Coquette, France). Chromogenic detection was carried out using DAB chromogen kit (Dako, Saint Aubin, France). Nuclei were counterstained with hematoxylin. Quantitative expression of all immunostainings was performed using positive pixels algorithm (Indica Labs, Corrales, NM). Results are expressed as percentage of positive pixels. The quantification method is an automated observer-independent process based on section scanning and application of publicly available algorithms. All images were acquired on an Axiovert 200 M microscope (Zeiss, Marly-le-Roi, France).

### Metabotyping of liver tissue by ^1^H NMR spectroscopy

Methods for sample preparation and ^1^H NMR spectra acquisition on a Bruker spectrometer (Rheinstetten, Germany) operating at 600.22 MHz frequency were as described previously^23^.

### Genetic linkage analysis

Genotyping was carried out with 1538 mouse single nucleotide polymorphism markers using the Illumina Golden Gate assay protocol (Illumina, Inc. San Diego, CA). The R ‘qtl’ package was used for linkage analysis using a genetic map constructed in the cross (Supplementary Table S3) as previously described in the same cross^12^. Grades of liver histopathology (micro- and macrovascular lesions, inflammation, ballooning, necrosis) were used as quantitative trait for genetic analyses, whereas continuous values of glycemia, organ and body weights and liver triglyceride concentrations were used for QTL mapping. Spectral peaks of ^1^H NMR metabolic profiles from liver extracts were aligned and quantified using methods previously optimized in the cross^12,24^ in order to test genetic linkage between quantitative values of metabolites and SNP markers across the mouse genome.

### RNA isolation and quantitative RT-PCR

RNA was extracted from liver using RNeasy RNA Mini Kit (Qiagen, Courtaboeuf, France). Reverse transcription was performed from a 20 μL reaction mixture with 500 ng RNA using M-MLV reverse transcriptase kit (ThermoFisher, Villebon, France). Quantitative RT-PCR was performed using sequence specific primers and MESA green kit for SYBR green assays (Eurogentec, Angers, France). We used the cyclophilin housekeeping gene to normalize relative quantification of mRNA levels using the Livak and Schmittgen methods^25^. Oligonucleotide sequences are listed in Supplementary Table S4.

## Supplementary information

Supplementary Materials
