# Supplementary material for: Systems Genetics of Hepatic Metabolome Reveals Octopamine as a Target for Non-Alcoholic Fatty Liver Disease Treatment
Source: Sci Rep. 2019 Mar 6;9:3656. doi: 10.1038/s41598-019-40153-0 (PMC6403227; doi:10.1038/s41598-019-40153-0)
Supplement: Supplementary file 1 — Supplementary Materials [file 41598_2019_40153_MOESM1_ESM.pdf]

Systems Genetics of Hepatic Metabolome Reveals Octopamine as a Target for Non-Alcoholic Fatty Liver Disease Treatment

Francois Brial, Aurélie Le Lay, Lyamine Hedjazi, Tsz Tsang, Jane F Fearnside, Georg W Otto, Fawaz Alzaid, Steven P Wilder, Nicolas Venteclef, Jean-Baptiste Cazier, Jeremy K Nicholson, Chris Day, Alastair D Burt, Ivo G Gut, Mark Lathrop, Marc-Emmanuel Dumas, Dominique Gauguier

Supplementary Materials

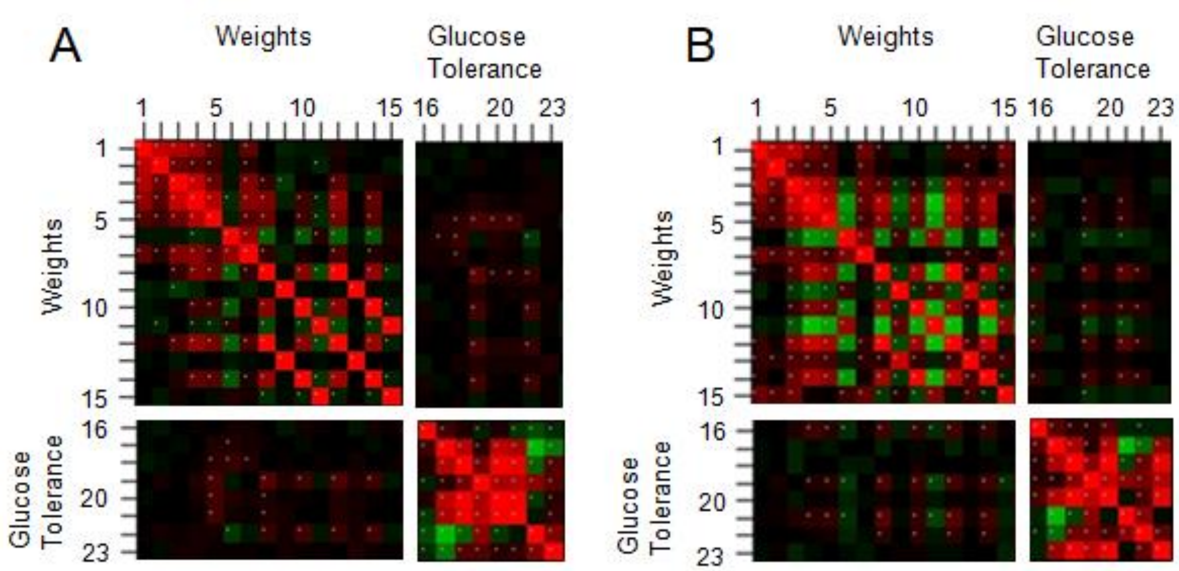

Supplementary Figure S1. Correlation matrices for phenotypes measured in 129S6xBALB/c F2 mice on control carbohydrate diet (A) and on high fat diet (B).

Positive correlations are shown in red and negative correlations in green . White dots indicate pointwise significance <0.05.

1: Body weight at 2 month; 2: Body mass index at 2 month; 3: Body weight at 3 month; 4: Body weight at 5 month; 5: Body mass index at 5 month; 6: Ratio liver weight to body weight; 7: Liver weight ; 8: Adiposity index (epidydymal fat pads); 9: Ratio brown adipose tissue weight to body weight; 10: Adiposity index (retroperitoneal fat pads); 11: Ratio heart weight to body weight; 12: Adiposity tissue weight (epidydymal fat pads); 13: Brown adipose tissue weight; 14: Adiposity tissue weight (retroperitoneal fat pads); 15: Heart weight; 16: Glycemia before glucose injection; 17: Glycemia 15 minutes after glucose injection; 18: Glycemia 30 minutes after glucose injection; 19: Glycemia 75 minutes after glucose injection; 20: Cumulative glycemia; 21: K parameter (15-75 minutes); 22: K parameter (15-30 minutes); 23: DG.

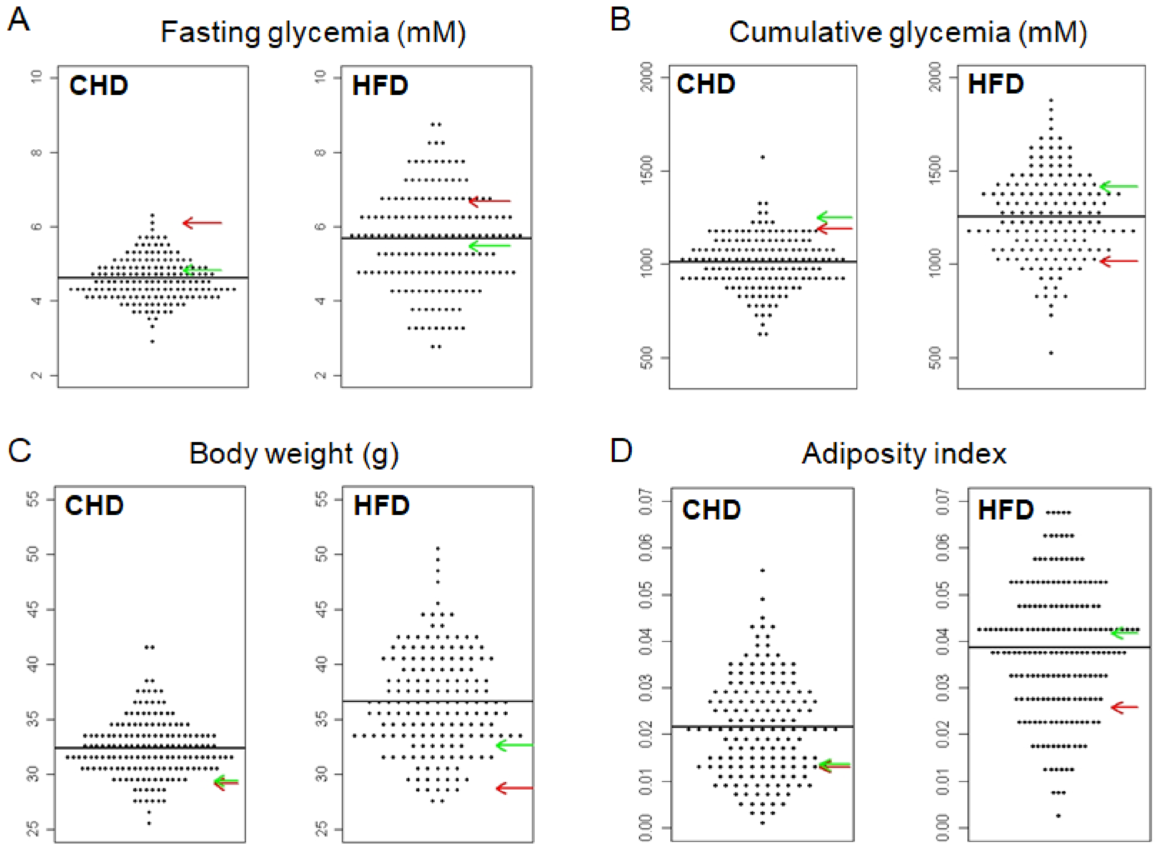

Supplementary Figure S2. High fat diet feeding in 129S6xBALB/c F2 mice increases phenotype variability. Distribution of fasting glycemia (A), cumulative glycaemia (B), body weight (C), adiposity index (D) are shown in F2 mice fed control (CHD) or high fat diet (HFD). Solid black line indicates phenotype means in the F2 cohort. Green and red arrows indicate mean values of corresponding phenotypes in 129S6 and BALB/c mice, respectively. Phenotype variability was markedly increased in 129S6xBALB/c F2 mice fed HFD when compared to F2 mice fed CHD.

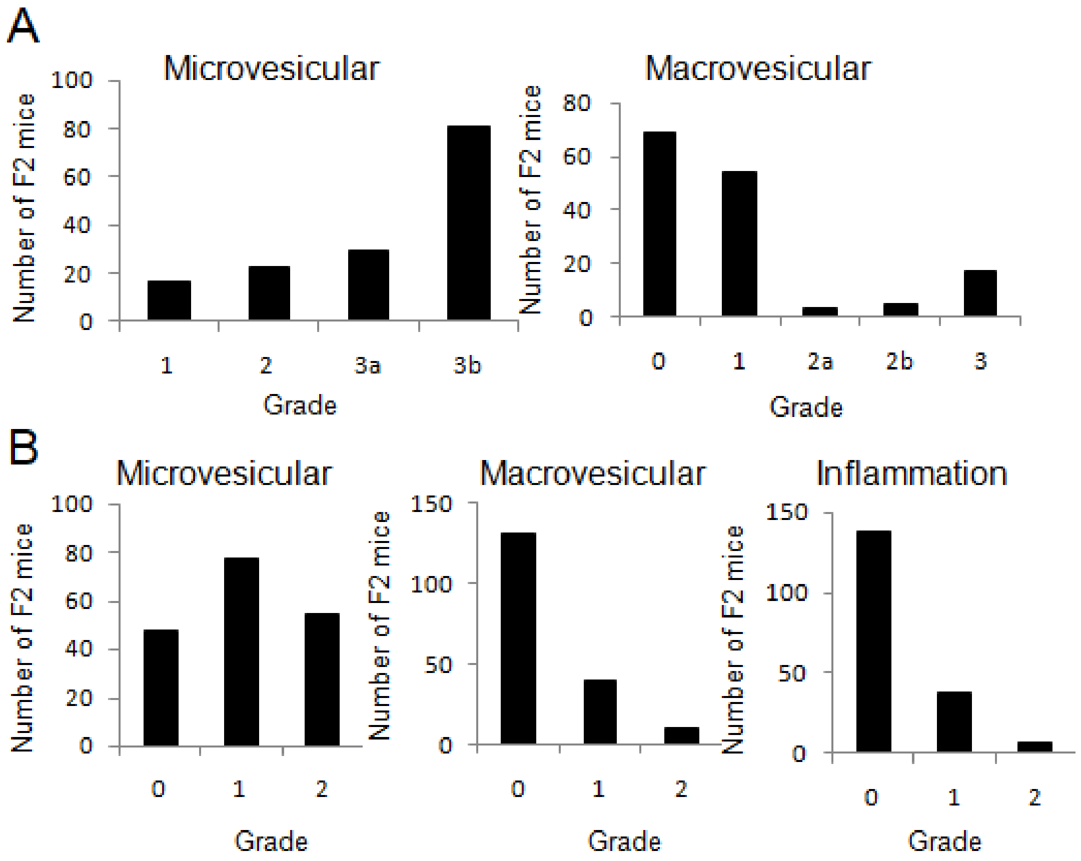

Supplementary Figure S3. Frequency of liver histopathology phenotypes in 129S6xBALB/c F2 mice fed either control (A) or high fat diet (B). At the age of 5 weeks, F2 mice were fed ad libitum either a standard carbohydrate chow or a 40% high fat diet. Liver histopathology was carried out in 5 months old F2 mice fed control diet (n=148) or high fat diet (ie. 15 weeks of high fat diet)(n=181).

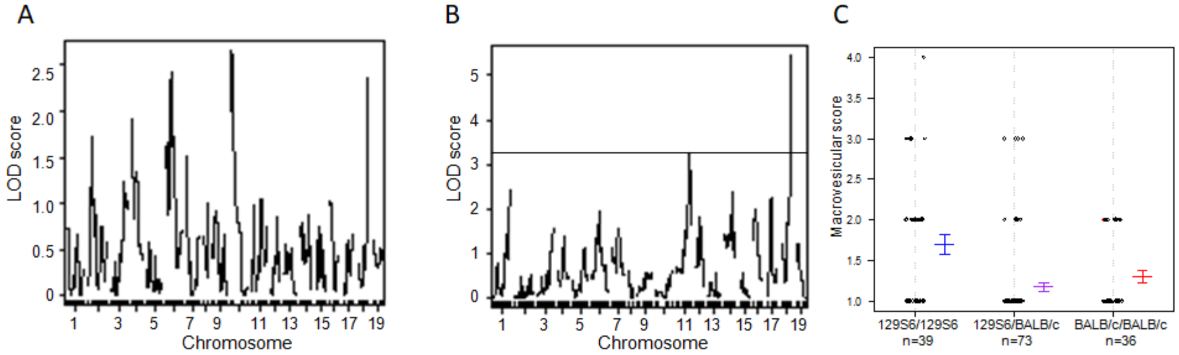

Supplementary Figure S4. Genome-wide linkage mapping of liver histopathology in 129S6xBALB/c F2 mice fed carbohydrate diet. Linkage mapping data for microvesicular (A) and macrovesicular lesions (B) in control-fed F2 mice are shown. LOD scores are plotted against map distances (centimorgans). A straight line in (B) indicates statistically significant LOD threshold (P=0.001). Effects of genotypes at the marker locus on chromosome 18 (C) exhibiting the strongest evidence of linkage to macrovesicular steatosis are illustrated by mean values (± SD) of the phenotypes calculated according to the genotype homozygous for the 129S6 or BALB/c alleles, or heterozygous at the locus.

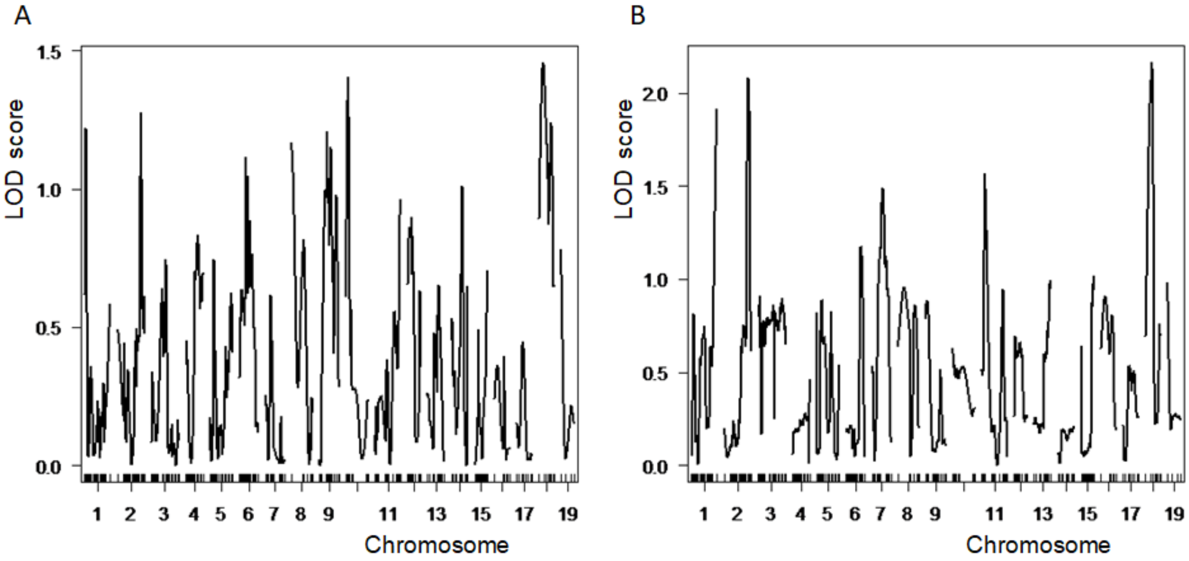

Supplementary Figure S5. Genetic analysis of liver necrosis (A) and ballooning (B) in high fat diet 129S6xBALB/c F2 mice. LOD scores are plotted against map distances (centimorgans).

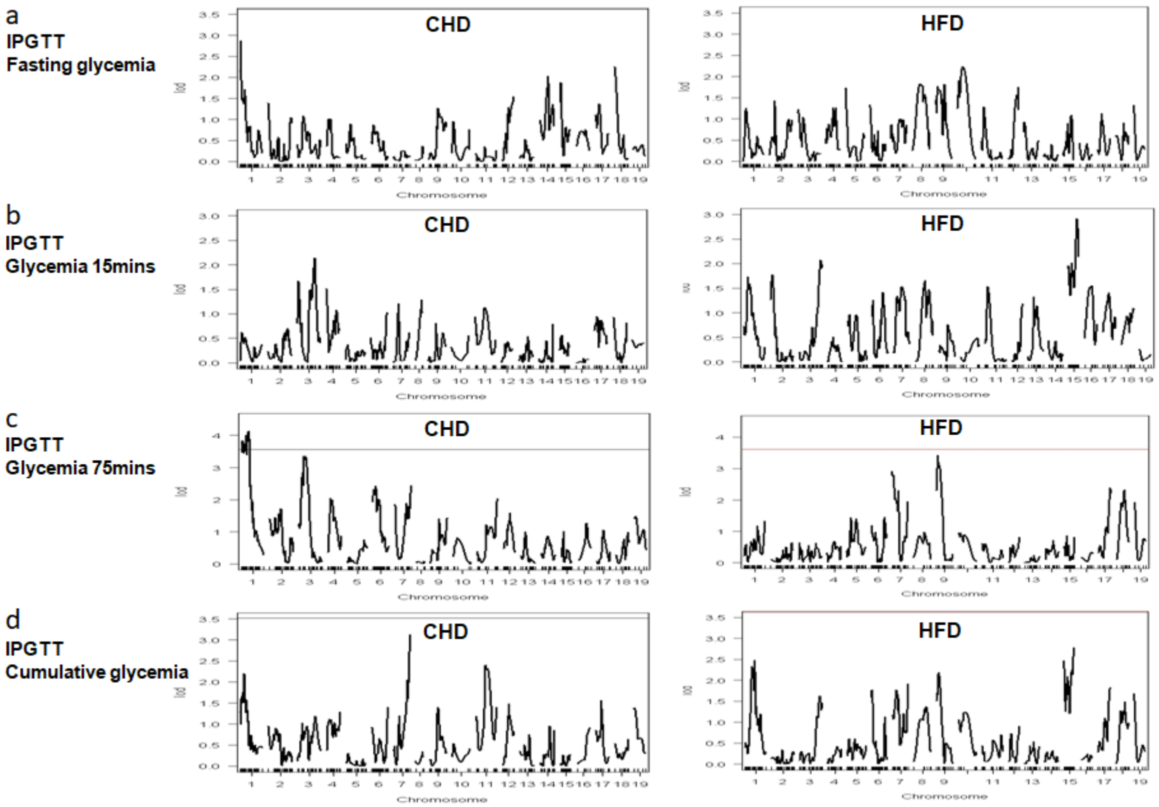

Supplementary Figure S6. Genome-wide scans for fasting glycemia (a), glucose response 15 (b) and 75 (c) minutes after glucose injection and cumulative glycemia during the IPGTT in (129S6xBLAB/c)F2 mice fed either control diet (CHD) or high fat diet (HFD). Solid lines indicate threshold of statistical significance.

Supplementary Table S1. Physiological characteristics of 129S6 x BALB/c F2 mice fed carbohydrate diet (CHD; n=148) or high fat diet (HFD; n=181). Data are mean ± SEM. * $P<0.05$; ** $P<0.001$ significant differences between F2 mice fed HFD and CHD.
EPD: Epidydimal fat pad. IPGTT: Intraperitoneal Glucose Tolerance Test.

**Phenotype**	**CHD**	**HFD**
Body weight (5 weeks) (g)	20.74 ± 0.20	21.41 ± 0.13
Body weight (20 weeks) (g)	32.33 ± 0.20	36.51 ± 0.35 **
Body mass index (20 weeks)	3.03 ± 0.02	3.26 ± 0.33 **
EPD weight (g)	0.62 ± 0.03	1.25 ± 0.04 **
Adiposity index (x1000)	21.49 ± 0.83	38.35 ± 1.04 **
Digestible energy (mJ/Kg)	53.16 ± 1.18	56.55 ± 2.55
Fasting glycemia (mM)	4.68 ± 0.04	5.68 ± 0.10 **
IPGTT, glycemia 15mins (mM)	16.86 ± 0.20	17.24 ± 0.26
IPGTT, glycemia 30mins (mM)	17.46 ± 0.19	17.46 ± 0.26
IPGTT, glycemia 75mins (mM)	9.11 ± 0.16	9.9293 ± 0.22 *
IPGTT Cumulative glycemia (mM)	1017 ± 10	1254 ± 17 **

Supplementary Table S2. Genetic loci significantly linked to liver 1H-NMR metabolomic features in high fat diet fed mice of the (129S6x BALB/c)F2 cross. Potential candidate metabolites at given chemical shift resonance are given. Chr, chromosome; cM, centiMorgan.

Chr	cM	Marker	Min	Max	ppm	Lod	Potential candidate metabolites
1	47.2	rs13476036	4.159	4.164	4.1615	4.72	-
3	23.6	rs13477108	0.85185	0.8612	0.856525	5.33	Cholesterol C25 C26 / Lipid CH3
3	23.6	rs134771081	1.2781	1.288	1.28305	5.87	Isoleucine / Lipid CH2CH2CH2CO
3	23.6	rs134771082	2.0822	2.0872	2.0847	4.91	Glutamate/Glutamine
3	23.6	rs134771083	2.4227	2.4331	2.4279	4.53	Carnitine / Alpha-ketoglutarate
3	23.6	rs3719360	3.9071	3.922	3.91455	3.98	Creatine
3	23.6	rs37193601	4.197	4.2058	4.2014	3.91	D(-)-3-Phosphoglycerate / o-Phosphoserine / p-Hydroxyphenyllacate
3	23.6	rs37193602	6.1747	6.1836	6.17915	6.23	-
3	23.6	rs37193603	7.9413	7.9512	7.94625	4.74	-
3	23.6	rs134771084	8.5513	8.5601	8.5557	6.18	-
3	25	c3.loc25	0.72975	0.7391	0.734425	3.92	-
3	25	c3.loc251	2.4337	2.4441	2.4389	3.96	Carnitine / Alpha-ketoglutarate
3	25	c3.loc252	4.0017	4.0303	4.016	3.83	-
3	25	c3.loc253	8.273	8.279	8.276	4.05	-
3	25	c3.loc254	8.5326	8.5486	8.5406	4.43	-
3	30	c3.loc30	0.8656	0.87495	0.870275	5.09	-
3	30	c3.loc301	8.7234	8.7295	8.72645	3.94	Nicotinurate
4	23.9	CEL.4_95976899	2.6669	2.6718	2.66935	3.91	Acetylcarnitine
4	27.3	rs13477899	2.6509	2.6575	2.6542	4.05	Acetylcarnitine
4	27.3	rs134778991	2.9259	2.9353	2.9306	3.93	-
5	6.5	rs3697291	9.593	9.5991	9.59605	4.43	-
6	5.1	rs6182329	7.085	7.0927	7.08885	3.90	-

8	59	D8Mit291	2.5871	2.597	2.59205	4.27	-
8	60	c8.loc60	2.5118	2.5365	2.52415	3.83	-
8	65	c8.loc65	1.0361	1.057	1.04655	4.28	Branched chain amino acids
9	40	c9.loc40	1.0361	1.057	1.04655	3.82	Branched chain amino acids
10	0	D10Mit123	1.0697	1.0746	1.07215	3.91	Alpha-ketobutyrate
10	5.4	rs6378338	3.218	3.2268	3.2224	4.50	Acetylcholine / Homoserine / Carnitine
12	7.9	rs3724198	4.3636	4.3702	4.3669	4.00	Uridine
12	7.9	rs37241981	8.1184	8.125	8.1217	4.10	N-Formylglycine
12	7.9	rs37241982	8.207	8.2125	8.20975	4.18	Adenine
12	7.9	rs37241983	8.5755	8.5854	8.58045	4.10	Purine
12	25	c12.loc25	3.2757	3.2839	3.2798	3.89	Betaine / Taurine / TMAO
13	0.6	rs6307428	2.1977	2.2027	2.2002	6.13	Adipate
13	25.7	rs13481825	8.1949	8.2037	8.1993	3.86	Adenine
17	19.7	rs3682923	2.5976	2.6036	2.6006	4.46	-
17	20.8	rs13482999	7.5871	7.5965	7.5918	3.98	N-formyktryptophan / Nicotinurate
17	20.8	rs134829991	7.597	7.6031	7.60005	4.80	N-formyktryptophan / Nicotinurate
17	25	c17.loc25	0.6907	0.6962	0.69345	4.05	Cholesterol C18
17	25	c17.loc251	2.5871	2.597	2.59205	4.28	-
17	25	c17.loc252	4.2091	4.214	4.21155	3.93	-
17	25	c17.loc253	4.2635	4.2701	4.2668	4.97	-
17	25	c17.loc254	8.2796	8.2922	8.2859	3.88	Nicotinate
17	25	c17.loc255	8.5046	8.5216	8.5131	4.20	-
17	30	c17.loc30	0.8656	0.87495	0.870275	4.12	Lipid CH3CH2CH2C=
17	30	c17.loc301	4.4857	4.4945	4.4901	3.98	1-Methylnicotinamide / Homocarnosine / Anserine
17	30	c17.loc302	6.0477	6.0543	6.051	4.05	-
17	30.3	rs3703241	6.1659	6.172	6.16895	3.91	-
18	25	c18.loc25	7.3385	7.3452	7.34185	4.31	Octopamine

Supplementary Table S3. General features of the genetic maps constructed in 129S6xBALB/c F2 mice. Published genetic lengths of the mouse chromosomes (cM) are as from Shifman *et al* 2006

Chrom	Markers	Genetic length (cM)		Average spacing (cM)
		Published	Calculated	
1	47	118	92	3.5
2	44	108	98	2.8
3	32	90	99	4
4	20	102	62	6.8
5	29	107	82	4.2
6	33	90	69	3.1
7	15	90	69	5.9
8	9	81	76	11.5
9	28	86	72	5.1
10	10	83	80	10.4
11	17	97	93	6.9
12	17	69	46	5.3
13	16	70	62	4.3
14	26	60	59	3.1
15	25	65	46	3.6
16	6	63	55	12.6
17	15	63	55	5.3
18	11	64	54	5.8
19	4	54	53	13.5
Total	404	1560	1322	

Supplementary Table S4. Oligonucleotides used for quantitative RT PCR in octopamine treated mice.

Gene	Forward	Reverse
Col1	CACCCCAGCGAAGAACTCATA	GCCACCATTGATAGTCTCTCCTAAC
Col3	GCACAGCAGTCCAACGTAGA	TCTCCAAATGGGATCTCTGG
Cyclophilin A	ATGGCACTGGCGGCAGGTCC	TTGCCATTCCTGGACCCAAA
Hsl	AGACACCAGCCAACGGATAC	ATCACCCTCGAAGAAGAGCA
Il10	GGTTGCCAAGCCTTATCGGA	ACCTGCTCCACTGCCTTGCT
Il6	GCTGGAGTCACAGAAGGAGTGGC	TCTGACCACAGTGAGGAATGTCCA
Pnpla2	ACAGTGTCCCCATTCTCAGG	TTGGTTCAGTAGGCCATTCC
Tnf	AGCCCACGTCGTAGCAAACC	GAGGAGCACGTAGTCGGGGC
